# North-South disparities in English mortality1965–2015: longitudinal population study

**DOI:** 10.1136/jech-2017-209195

**Published:** 2017-08-07

**Authors:** Iain E Buchan, Evangelos Kontopantelis, Matthew Sperrin, Tarani Chandola, Tim Doran

**Affiliations:** 1 Farr Institute, Faculty of Biology Medicine and Health, University of Manchester, Manchester, UK; 2 Manchester Academic Health Science Centre, University of Manchester, Manchester, UK; 3 NIHR Manchester Biomedical Research Centre, Central Manchester Foundation NHS Trust, Manchester, UK; 4 School for Primary Care Research, Radcliffe Observatory Quarter, National Institute for Health Research, Manchester, UK; 5 School of Social Sciences, University of Manchester, Manchester, UK; 6 Department of Health Sciences, University of York, York, UK

**Keywords:** inequalities, mortality, geography

## Abstract

**Background:**

Social, economic and health disparities between northern and southern England have persisted despite Government policies to reduce them. We examine long-term trends in premature mortality in northern and southern England across age groups, and whether mortality patterns changed after the 2008–2009 Great Recession.

**Methods:**

Population-wide longitudinal (1965–2015) study of mortality in England's five northernmost versus four southernmost Government Office Regions – halves of overall population. Main outcome measure: directly age-sex adjusted mortality rates; northern excess mortality (percentage excess northern vs southern deaths, age-sex adjusted).

**Results:**

From 1965 to 2010, premature mortality (deaths per 10 000 aged <75 years) declined from 64 to 28 in southern versus 72 to 35 in northern England. From 2010 to 2015 the rate of decline in premature mortality plateaued in northern and southern England. For most age groups, northern excess mortality remained consistent from 1965 to 2015. For 25–34 and 35–44 age groups, however, northern excess mortality increased sharply between 1995 and 2015: from 2.2% (95% CI –3.2% to 7.6%) to 29.3% (95% CI 21.0% to 37.6%); and 3.3% (95% CI –1.0% to 7.6%) to 49.4% (95% CI 42.8% to 55.9%), respectively. This was due to northern mortality increasing (ages 25–34) or plateauing (ages 35–44) from the mid-1990s while southern mortality mainly declined.

**Conclusions:**

England's northern excess mortality has been consistent among those aged <25 and 45+ for the past five decades but risen alarmingly among those aged 25–44 since the mid-90s, long before the Great Recession. This profound and worsening structural inequality requires more equitable economic, social and health policies, including potential reactions to the England-wide loss of improvement in premature mortality.

## Introduction

England has profound and persistent regional divides in economy, society and health.^1–3^ Major structural disparities in power and resources between northern and southern territories were described as far back as the 11th century in the wake of Norman suppression of the North.^4^ By the 18th century the concept of a North-South divide, with political and economic power concentrated in the South, was widely acknowledged^5^ and judged unacceptable by commentators from across the political spectrum. In 1845, the plight of the working classes following an extended period of rapid industrialisation was placed in the context of regional differences in material conditions and health by future Prime Minister Benjamin Disraeli in his novel *Sybil*—*The Two Nations*,^6^ and by Friedrich Engels in *The Condition of the Working Class in England*,^7^ leading to the development of radically different political solutions.^8^ In more recent political history, successive governments have commissioned reports on health inequalities and have set policies to reduce them,^9–12^ but the divide has remained.

In 2010, we used the historical geography of the North-South divide to examine trends over time in English mortality and found a persistent 20% northern excess in premature (under age 75 years) mortality from 1965 to 2008.^13^ Over this period, the total burden of mortality in England fell by around 50% in men and 40% in women, but the North-South gap did not close. For younger age groups (under 50 years), the gap *increased* from the mid-1990s onwards, despite a relatively benign economic climate and the implementation of national policies to reduce inequalities in health. This worsening of early premature mortality remains underexplored.

The Great Recession of 2008–2009 raises new questions about inequalities in mortality. Somewhat counterintuitively, mortality rates tend to decline faster during economic downturns, mediated in part by changes in: work and leisure patterns (through greater available time for family, leisure and physical activities and a reduction in motor vehicle deaths); and health-related behaviours (through a reduction in risky health behaviours during recessions).^14^ Some adverse risky behaviours such as excessive alcohol use decline during recessions and increase during periods of economic growth.^15^ However, the impact on inequalities in mortality is less clear. Higher levels of deprivation in the North may result in a slower decline in mortality rates during recessions. In addition, the depth of the 2008–2009 recession was greater than previous recessions in 1990–1991, 1980–1981 and 1973–1975, and it was followed by a prolonged period of austerity and restricted public spending. During this period, the long-term trend for declining suicide rates sharply reversed in the UK, with the greatest increases occurring in the areas experiencing the highest rises in unemployment^16^; areas concentrated in the North.^17^ Given the strong associations between unemployment, (relative) poverty, social welfare and health, it is reasonable to consider how this economic shock and the subsequent policy response impacted on long-standing regional disparities in mortality.^18^ The current UK/global political and economic turbulence raises the importance of new policy evidence in this area.

In the present study, we identify the age groups most affected by northern excess mortality and examine long-term trends in mortality for these groups in the North and the South. We also examine changes in these long-term trends following the 2008–2009 Great Recession.

## Methods

### Data sources and geographies

For the whole of England and by region, we obtained annual counts of deaths (by any cause, registered in the specified year), and midyear population estimates in all years from 1965 to 2015, from the Office for National Statistics (ONS). The ONS midyear populations were based on Decennial Censuses and interpolated estimates obtained for the remaining years (extrapolated for 2012 onwards). The death and population data were stratified by sex and age group (infants; 1–4; 5–9; …; 80–84; 85 and over). We also used ONS deprivation statistics: Index of Multiple Deprivation 2015 at lower layer super output area (districts of mean 1500 population).

The regions were defined, using established methodology,^13^ as aggregates of Government Office Regions: North (North East, North West, Yorkshire and The Humber, East Midlands and West Midlands); and South (East, South West, London and South East). This geography divides the English population into two approximately equal halves: the five northernmost and the four southernmost Government Office Regions, which approximates to a line drawn between the Wash and the Severn Estuary, and is consistent with previous studies.^4 19 20^

### Statistical analyses

Mortality rates were calculated from death registrations and midyear population estimates and examined by age group, sex and region (North vs South England) for each year from 1965 to 2015.

Age-sex adjusted mortality rates were derived using direct standardisation for population structure, with the total period population as the reference, and were used for comparing changes over time by regions and age groups.

Where the only statistic drawn from each year was a mortality rate ratio, Poisson models were fitted by year (with death as the outcome, the natural logarithm of the midyear population size estimate as the offset, and age/sex groups as categorical covariates). In this way, North and South were compared after standardising for age and sex across the entire population within the relevant year, and the adjusted mortality rate ratios were presented as ‘northern excess mortality’ (the percentage of excess deaths in the North compared with the South after adjusting for differences in age and sex).

To study how age-specific mortality rate ratios between North and South changed over time, we fitted a Poisson model as above, but with age/sex/year groups and north/south status as categorical covariates. We included a three-way interaction between age group, year and north/south status.

To address the hypothesis of change around recession periods we report, from the Poisson model, relative change in overall northern excess premature mortality (under 75) in the year after the recession compared with 3 years earlier, for the recessions of 1973–1975, 1980–1981, 1990–1991 and 2008–2009.

Statistics are presented as the main effect with 95% CIs.

## Results

### Demographics

The average North:South population size ratio from 1965 to 2015 was 1.050 for men and 1.030 for women. The South grew faster than that of the North over the study period and by 2015 the North:South population ratios were 0.878 for men and 0.879 for women.

Changes in the age-sex structures of North and South England from 1965 to 2015 are illustrated in Supplementary figure A1. In the North, the median age for women increased from 37.0 in the first 3 years of the period to 41.4 in the last 3 years, and for men from 34.0 to 39.5. In the South, median ages increased from 38.4 to 40.8 for women and from 34.9 to 38.9 for men. Over the study period, the proportion of people over 65 increased more in North England (22.9% to 36.7%) than in South England (25.9% to 34.4%). Correspondingly, the proportion of men aged 25–44 decreased in the North (from 53.9% to 50.7%) but increased in the South (from 49.6% to 49.8%); for women, the proportions increased from 53.5% to 56.7% in the North and from 49.1% to 55.2% in the South.

10.1136/jech-2017-209195.supp1 Supplementary file 1

**Figure 1:**
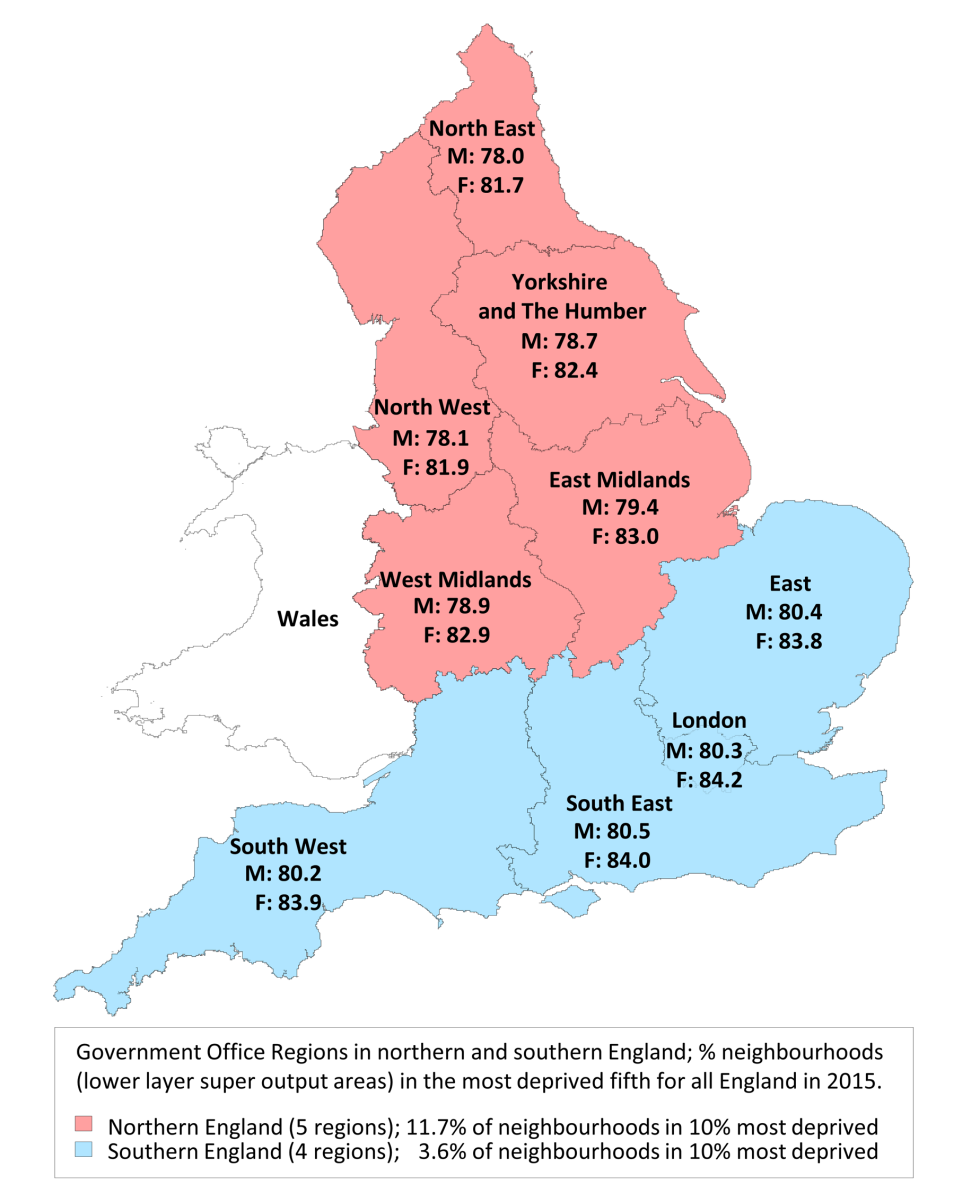
Map of England by Government Office Regions in northern and southern halves of population, showing expectation of life at birth for men and women for the years 2012–2014, with a summary of regional deprivation in 2015. F, females; M, males.

Figure 1 illustrates the split of English regions into northern and southern halves by expectation of life at birth (men, women) for each Government Office Region, and by material deprivation using the Index of Multiple Deprivation 2015 summarised as the proportion of neighbourhoods (lower layer super output areas: mean population size 1500) falling in the 10% most deprived for all England.

### Premature mortality time trends

**Figure 2:**
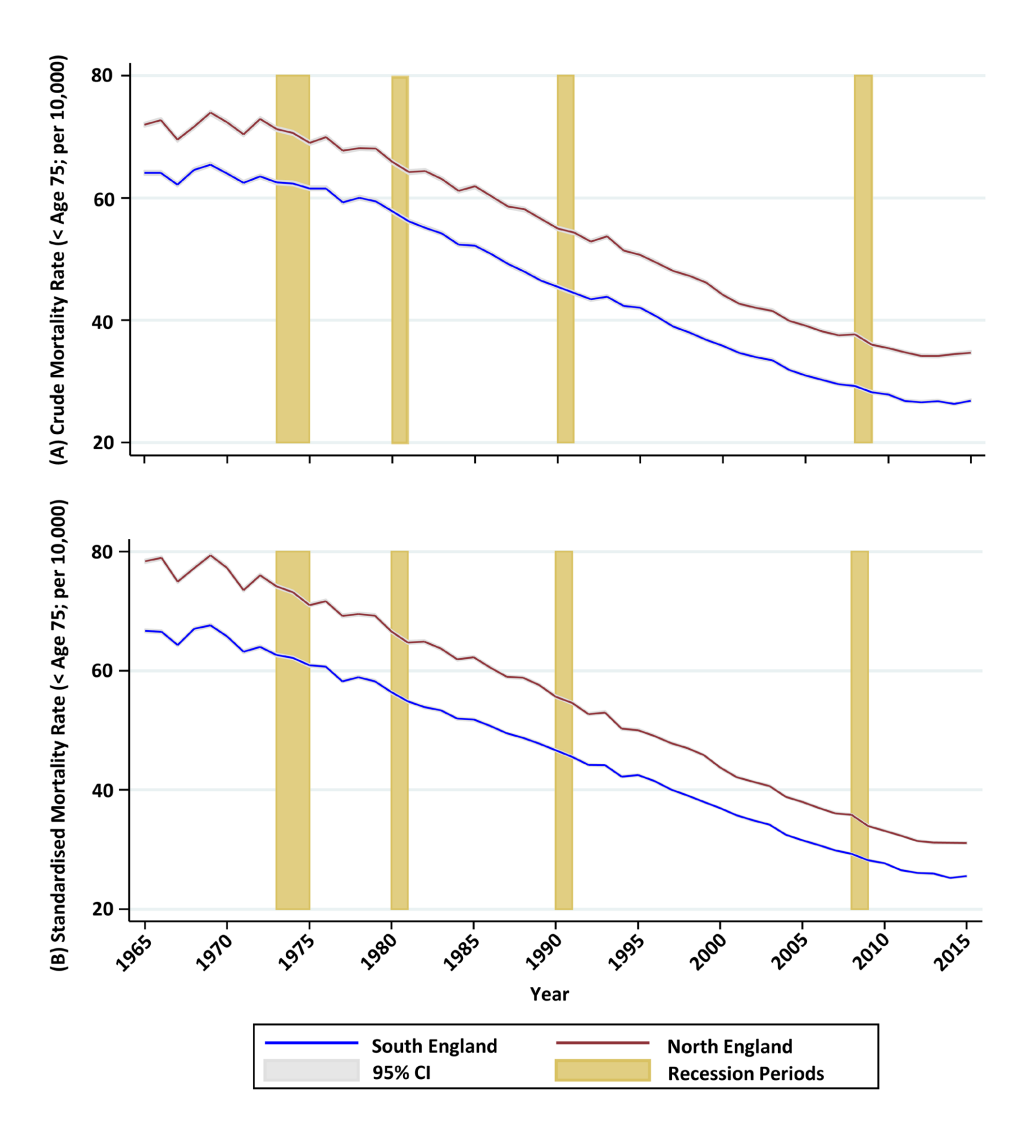
Premature (age <75 years) mortality for men plus women in the North and South of England, 1965–2015: (A) crude mortality rate; (B) directly age-standardised rate (using total period standard population).

Taking the key public health indicator of premature mortality, crude rates (as deaths per 10 000 population under age 75 years) fell in the North from 72 in 1965 to 35 in 2010 and in the South from 64 to 28 (figure 2A). From 2012, both crude and age-sex adjusted (figure 2B) rates appear to plateau. Relative changes in northern excess premature mortality before and after each recession were small (Supplementary table A1 and figure A2).

A gap in crude premature mortality rates of between 7.1 (1968) and 10.2 (1988) deaths per 10 000 aged <75 years, changing smoothly over time, existed between North and South throughout the study period (figure 2A). In 1965, the difference in age-sex adjusted rates was 11.9 per 10 000, steadily closing thereafter to 5.6 per 10 000 in 2010 (figure 2B). After 2010, this gap in crude rates widened while the rise in age-sex adjusted relative rates became larger.

After adjusting within each year for the age-sex population structure differences between North and South England, using Poisson models, northern excess premature mortality increased from 17.5% (95% CI 16.7% to 18.4%) in 1965 to 21.5% (95% CI 20.2%–22.7%) in 2015, with the largest excess being 23% (95% CI 21.8% to 24.3%) in 2014 (Supplementary figure A2). In absolute terms, the total number of northern excess premature deaths throughout the study period was 1 173 360 (95% CI 1 112 724 to 1 234 280).

### Age-group specific mortality time trends

**Figure 3:**
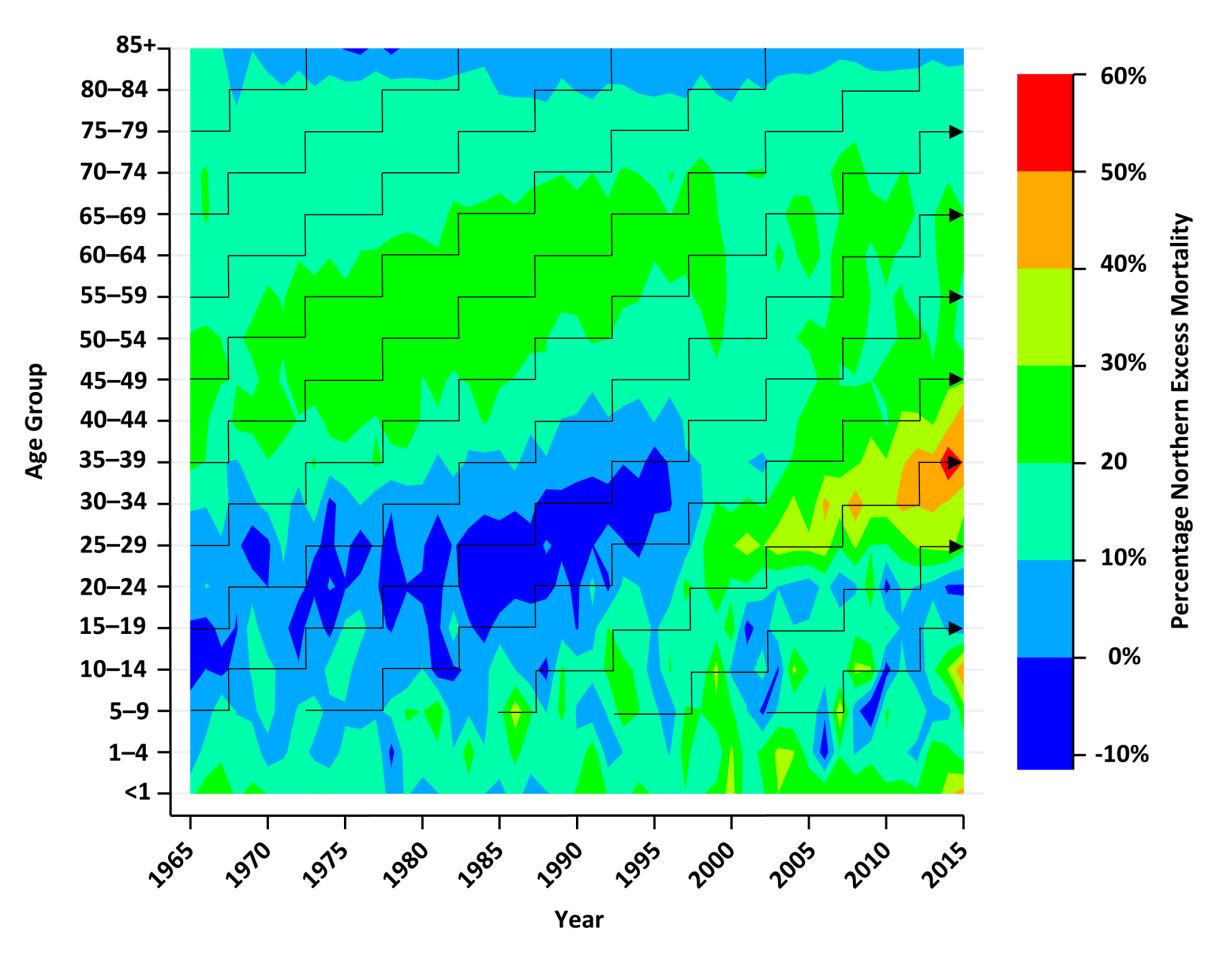
Excess mortality in the North compared with South of England by age groups in a Lexis diagram from 1965 to 2015.

Figure 3 shows the northern excess mortality for all age groups and years in this study. The current hot spot of over 50% greater risk of death in middle adult life in the North compared with the South appears to have a cohort pattern originating in the mid-90s. We do not make strong inferences about younger age groups where numbers of deaths are small. The following analyses unpick mortality trends over time in wider age bands.

**Figure 4:**
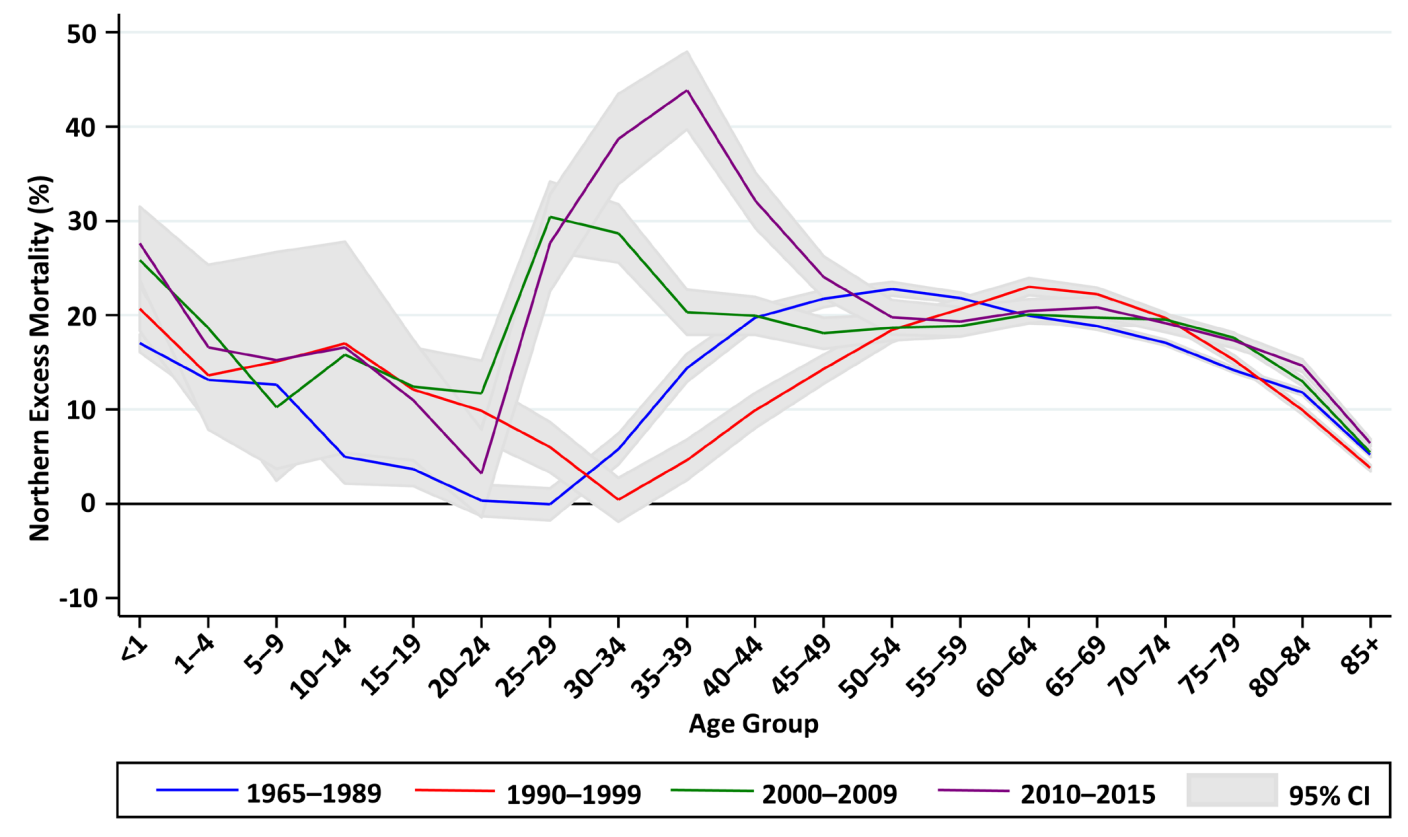
Excess mortality in the North compared with South of England by age group in four periods between 1965 and 2015.

Figure 4 examines excess mortality in the North by age group, using Poisson models with age-group/year/region interaction terms, contrasting the 2010–2015 period following the Great Recession with the two previous decades, and with the early half of the study period. Over five decades, northern excess mortality followed a consistent pattern: high (13%–25% excess) in the under 5s, declining with increasing age to the mid-30s, then climbing to a peak (18%–23% excess) in the 50–74 age group, before falling again. Prior to 2000, the lowest northern excess mortality was consistently found in the 20–34 age group, but after 2000 the pattern reversed with the 25–44 age group showing the highest northern excess mortality.

**Figure 5:**
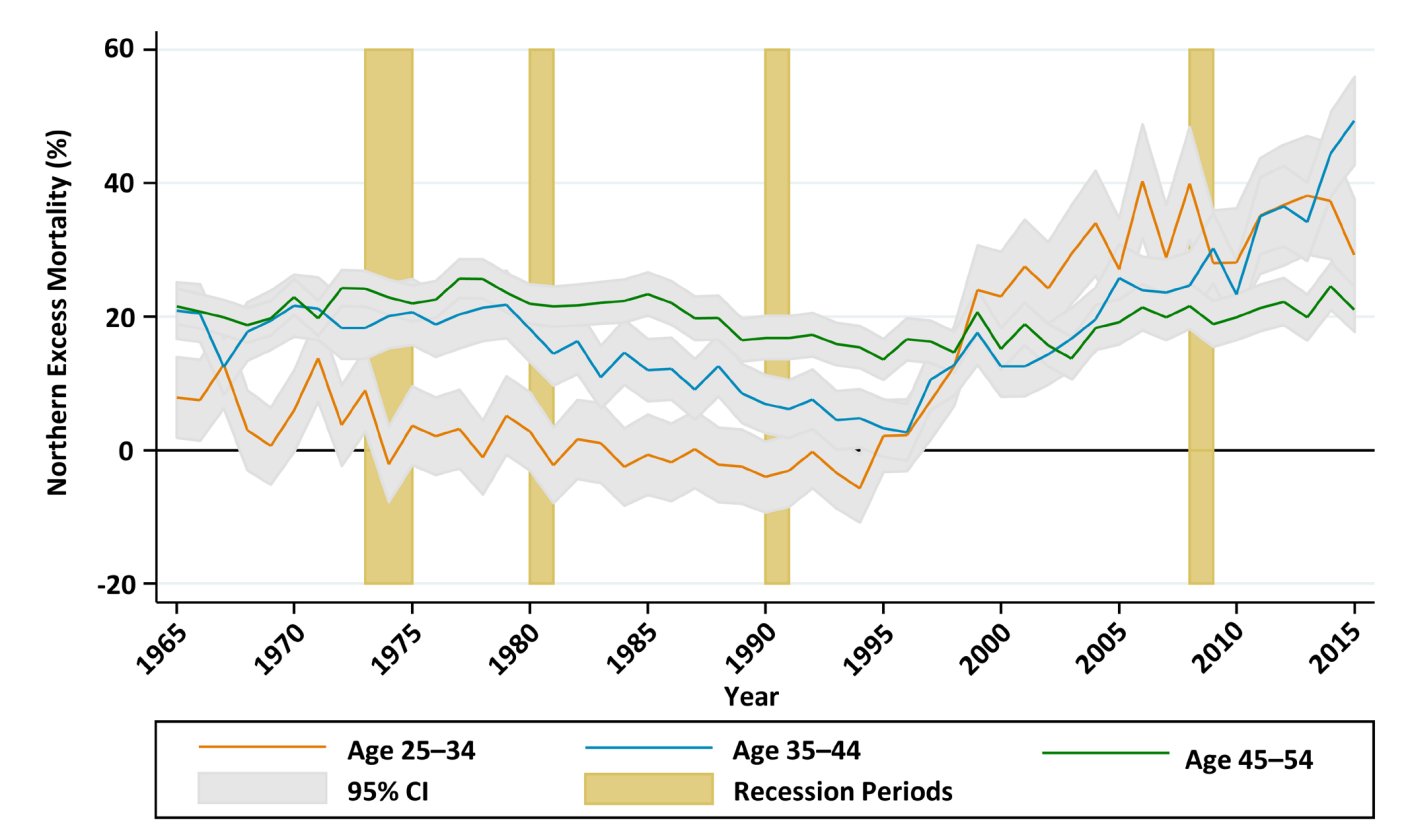
Excess mortality in the North compared with South of England for age groups 25–34, 35–44 and 45–54, from 1965 to 2014, adjusted for within-group age-sex differences.

**Figure 6:**
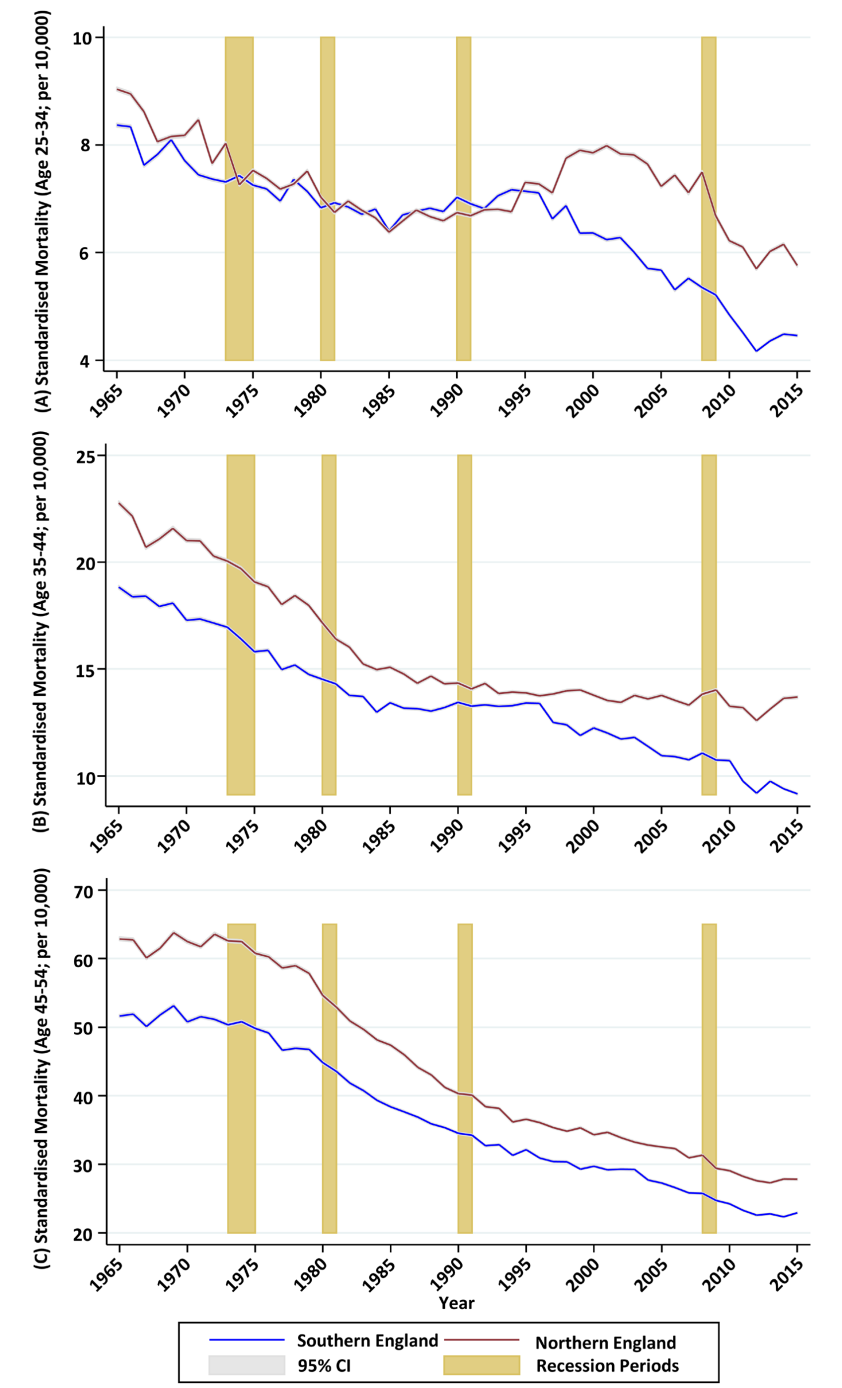
Directly age-standardised mortality rates (using total period standard population), North and South of England, 1965–2015; (A) age groups 25–34; (B) age groups 35–44; (C) age groups 45–54.

Important underlying patterns are seen by examining the change over time in age subgroup mortality in North and South England. Figure 5 charts the change over time in northern excess mortality at ages 25–34, 35–44 and 45–54 years, derived from within-year Poisson models:

- For the population aged 25–34, age-sex adjusted mortality rates declined in both the North and the South between 1965 and 1984, with a slightly faster rate of decline, from a higher baseline, in the North. Rates then climbed slightly in both regions until 1995, after which they continued to increase for the rest of the decade in the North while declining rapidly in the South, opening a large gap in excess mortality which persisted to the end of the study period. The absolute gap in age-sex adjusted mortality for the 25–34 age group (figure 6A) closed from 7.9% (95% CI 1.9% to 14.0%) in 1965 to 2.2% (95% CI −3.2% to 7.6%) in 1995 before increasing to 29.3% (95% CI 21.0% to 37.6%) in 2015.
- For the population aged 35–44, there was a similar pattern of declining mortality (steeper in the North) up to the mid-1980s, followed by plateauing mortality up to the mid-1990s. From 1995, age-sex adjusted mortality rates started to decline again in the South, while continuing to plateau in the North. The gap remained consistent between 1965 and 1979, before gradually disappearing by 1996. The absolute gap in age-sex adjusted mortality for the 35–44 age group (figure 6B), closed from 20.9% (95% CI 16.7% to 25.1%) in 1965 to 3.3% (95% CI −1.0% to 7.6%) in 1995, before increasing to 49.4% (95% CI 42.8% to 55.9%) in 2015.
- In contrast, the gap between North and South in the 45–54 age group (figure 6C) deviated relatively little from around 20% throughout the study period.

## Discussion

### Summary of findings

We examined long-term trends in mortality in the North and South of England, focusing on the age groups most affected by northern excess mortality. Between 1965 and 2010, national mortality declined while the northern excess remained consistent for most age groups. Among adults aged 25–44, however, northern excess mortality increased sharply from the mid-1990s onwards due to a stall in the North of the long-term decline in mortality. By 2012, following the Great Recession of 2008–2009, four decades of declining premature mortality in both North and South England had stalled, a pattern not seen following earlier recessions.

### Strengths and limitations of the study

This study analysed mortality data over five decades for England's population, enabling differences in long-term trends between North and South to be examined in full. Measuring inequalities between two large, meaningful geographies of around 25 million people each allowed us to minimise the effects of errors in population estimates, and more precisely to examine age group specific mortality. While the classification of smaller geographies into North or South is to some extent arbitrary, it reflects boundaries of administration and political responsibility, as well as established social, economic and cultural divisions. This dichotomous approach does not, however, allow us to account for heterogeneity within each region. Within the South for example, London concentrates political and economic resources. We have not examined causes of death, which limits the conclusions we can draw about the underlying drivers of the disparities, although the relative ranking of causes of death for different age groups is the same across all regions of the country.^21^ We also have not measured the changes over time in potential social, economic, behavioural and demographic determinants (eg, migration, smoking rates and unemployment), as this was beyond the scope of the study. Finally, in our analysis of recessionary periods we have not examined other periods of economic or social insecurity.

### Interpretation of findings

In common with other advanced industrial nations, mortality rates consistently fell across all regions of England in the second half of the 20th century and the first decade of the 21st. Premature mortality rates in the North steadily converged on—but remained substantially higher than—rates in the South throughout a period that included three recessions and multiple changes in political administration, with a total of 23 years of Labour and 22 years of Conservative government. However, the same uniform pattern of converging mortality was not apparent in all age groups. For the 25–34 and 35–44 age groups there were distinct phases: between 1965 and 1985, mortality rates fell more quickly (from a higher baseline) in the North, closing the North-South gap to 12% in the 35–44 age group and completely in the 25–34 age group. This was followed by a decade when the long-term decline in mortality rates among young adults halted nationwide, with little change in regional disparities. However, from the mid-1990s northern excess mortality increased by 25 percentage points in the 25–34 age group and by 23 percentage points in the 35–44 age group. Underlying this rapid divergence was an increase (ages 25–34) or plateauing (ages 35–44) of mortality rates in the North at the same time as mortality rates in the South started to decline again. Because deaths in the under 45s are relatively uncommon, these patterns are concealed in whole population mortality rates, which are dominated by older age groups. However, the consequences for the population, in terms of years of life lost, are grave.

Prior to the Great Recession of 2008–2009 there were no obvious effects of recessionary periods on either long-term trends in mortality or on inequalities in mortality between North and South, even in the working age population. For example, the divergence in mortality rates for young adults between North and South began in 1995, 4 years after the 1990–1991 recession, and continued for another decade through a period of sustained economic growth. It is possible, however, that overall mortality figures conceal different trends for specific causes of death; analyses of previous economic crises found that increases in cardiac deaths were counterbalanced by reductions in respiratory deaths.^22^

During the 2008–2009 recession, there was again no obvious change in underlying trends in mortality, but from 2012 the historical decline in under 75 mortality rates plateaued in both the North and the South. Within the under 75s, this overall pattern is reflected equally in North and South England for the 25–34 and 45–54 age groups; however, for the 35–44 age group the gap is widening due to a decline in mortality post-2012 not seen in the North. It remains to be seen whether the overall plateauing is a transient phenomenon, or whether years of improving premature mortality are fading and existing wide regional disparities are persisting or worsening.

The extended period of austerity following the 2008–2009 recession has raised concerns about detrimental impacts on population health,^23^ particularly the health of disadvantaged socioeconomic groups and more economically precarious regions.^18^ However, the divergent trends in mortality we noted in the 1990s and early 2000s suggest that inequalities can increase rapidly during periods of sustained economic growth. Similar patterns have emerged in the USA (and have been similarly concealed by overall mortality patterns), where mortality rates in white non-Hispanic women aged 45–54 started to increase in the late 1990s.^24^

Explaining the rapidly widening divide in young adult mortality between North and South over the past two decades will require detailed analysis of specific causes of death and the plausible explanations, including epidemiological, social, economic and migratory factors. The most common causes of death in this age group are suicide, poisoning, land transport accidents and liver disease. Over the age of 40, other causes become increasingly common; ischaemic heart disease in men and breast cancer in women.^21^ The recent rises in mortality rates noted above in the USA were driven by rapid increases in suicide, poisonings and chronic liver disease—increases that were heavily concentrated in less educated populations.^24^ In the UK, suicide rates declined nationally throughout the 1990s and 2000s, but this general fall may have concealed different regional and socioeconomic trends. The rapid increase in suicides from 2008, and its concentration in areas of high unemployment, suggests that already wide health inequalities affecting England are worsening.^18^ The age-standardised rate for alcohol-related deaths in the UK has increased since 1994. Although the rate peaked in 2008 and has subsequently fallen, the 2014 rate is still higher than that observed in 1994.^25^

Some commentators on the nation's growing inequalities conclude that the transition from premature mortality driven by infectious diseases in the Victorian era to chronic diseases today means that the era of effective state intervention—which included the great public works of sanitation, housing reform, immunisation and universal healthcare—is past, and that responsibility for addressing current disparities now lies with the individual.^26^ Following this logic, the government would do no more than provide education and some behavioural nudges, particularly in the case of the violent and self-destructive causes of premature mortality that afflict young and middle-aged adults. However, the strong social and geographical patterning of trends in premature mortality points to structural defects that lie beyond the control of the individual, demanding collective action and a strong policy response.

Future policies may be distracted from addressing inequalities due to the pan-regional nature of the apparent plateau in declining premature mortality and the reversal of declining elderly mortality.^27^ Doing so would ignore the persistent North-South divide and the potential population health gain from addressing it effectively.

## Conclusions

We have identified an alarming growth in England's North-South divide in mortality for the population aged 25–44, amid a persistent inequality accounting for 1.2 million northern excess deaths under age 75 over five decades. Policy responses to this profound, persistent health divide will reflect a variety of ideologies. Effective policies, however, may require substantial social and economic changes, including a rebalancing of the economy between North and South England that is proportionate to the scale of the problem.

**What is already known on this subject**

- From 1965 to 2008 the chances of dying early (age <75 years) were a fifth higher in the North of England than the South, while England's overall mortality fell by around 50% in men and 40% in women.
- Northern excess mortality decreased from the early 80s to the late 90s, then increased,with a large (22%) rise among the 20–34 years age group from 1996 to 2008.

**What this study adds**

- Since 1995, the North of England has experienced a profound rise in early (ages 25–44 years) premature mortality, relative to the South.
- Between 2010 and 2015, rates of premature (age <75 years) mortality have plateaued in both the North and South of England after falling for more than four decades.

## References

[1] Whitehead M, et al 2014 Due North Report of the Inquiry on Health Equity for the North: University of Liverpool and Centre for Local Economic Strategies www.cles.org.uk.

[2] : Baker ARH, Billinge M, Geographies of England, the North-South divide, imagined and material. Cambridge: Cambridge University Press, 2004.

[3] North SD South: Britain's Economic, Social and Political Divide. 2nd ed London: Penguin, 1994.

[4] Campbell BMS North South Dichotomies 1066–1550 In: Baker ARH, Billinge M, Geographies of England, the North-South divide, imagined and Material. Cambridge: cambridge University Press, 2004:145–74.

[5] Billinge M Divided by a common language 1750–1830 In: Baker ARH, Billinge M, Geographies of England, the North-South divide, imagined and Material. Cambridge: cambridge University Press, 2004:88–111.

[6] Disraeli B Sybil. Harmondsworth: Penguin, 1987.

[7] Engels F The condition of the Working Class in England. Harmondsworth: Penguin, 1987.

[8] Martin R The Political Economy of Britain's North-South Divide. Transactions of the Institute of British Geographers 1988;13:389–418. 10.2307/622738

[9] Black D 1980 Inequalities in Health: Report of a Research Working Group: Department of Health and Social Security.

[10] Acheson D, et al 1998 Independent Inquiry Into Inequalities in Health Report: Department of Health www.gov.uk/government/publications/independent-inquiry-into-inequalities-in-health-report.

[11] House of Commons Health Committee. 2009 Health Inequalities. Third Report of Session 2008–9: The Stationery Office www.publications.parliament.uk/pa/cm200809/cmselect/cmhealth/286/286.pdf.

[12] Marmot M, Allen J, Goldblatt P, et al 2010 Fair Society, Healthy Lives: Strategic Review of Health Inequalities in England Post 2010: UCL Institute of Health Equity www.instituteofhealthequity.org/projects/fair-society-healthy-lives-the-marmot-review.

[13] Hacking JM, Muller S, Buchan IE Trends in mortality from 1965 to 2008 across the English north-south divide: comparative observational study. BMJ 2011;342:d508 10.1136/bmj.d508 21325004 PMC3039695

[14] Bezruchka S The effect of economic recession on population health. CMAJ 2009;181:281–5. 10.1503/cmaj.090553 19720709 PMC2734206

[15] Ruhm CJ Healthy living in hard times. J Health Econ 2005;24:341–63. 10.1016/j.jhealeco.2004.09.007 15721049

[16] Barr B, Taylor-Robinson D, Scott-Samuel A, et al Suicides associated with the 2008-10 economic recession in England: time trend analysis. BMJ 2012;345:e5142 10.1136/bmj.e5142 22893569 PMC3419273

[17] Bell DNF, Blanchflower DG Uk unemployment in the great recession. Natl Inst Econ Rev 2010;214:R3–R25. 10.1177/0027950110389755

[18] Chetty R, Stepner M, Abraham S, et al The Association between Income and Life Expectancy in the United States, 2001-2014. JAMA 2016;315:1750–66. 10.1001/jama.2016.4226 27063997 PMC4866586

[19] Glasmeier A, Martin R, Tyler P, et al Poverty and place in the UK and USA. Cambridge J Regions Econ Soc 2008;1:1–16.

[20] Wells C, Gordon E Geographical variations in premature mortality in England and Wales, 1981-2006. Health Stat Q 2008;38:6–18. 18595384

[21] Office for National Statistics. Deaths Registered in England & Wales. 2015 www.ons.gov.uk/peoplepopulationandcommunity/birthsdeathsandmarriages/deaths/bulletins/deathsregisteredinenglandandwalesseriesdr/2015.

[22] Stuckler D, Meissner C, Fishback P, et al Banking crises and mortality during the Great Depression: evidence from US urban populations, 1929-1937. J Epidemiol Community Health 2012;66:410–9. 10.1136/jech.2010.121376 21441177

[23] Hiam L, Dorling D, Harrison D, et al Why has mortality in England and Wales been increasing? an iterative demographic analysis. J R Soc Med 2017;110:153–62. 10.1177/0141076817693599 28208027 PMC5407517

[24] Case A, Deaton A Rising morbidity and mortality in midlife among white non-Hispanic americans in the 21st century. Proc Natl Acad Sci U S A 2015;112:15078–83. 10.1073/pnas.1518393112 26575631 PMC4679063

[25] Office for National Statistics. ONS release on Alcohol Related Deaths in the United Kingdom. 2014 www.ons.gov.uk/peoplepopulationandcommunity/healthandsocialcare/causesofdeath/bulletins/alcoholrelateddeathsintheunitedkingdom/registeredin2014#time-trends-for-the-uk-as-a-whole-1994-to-2014.

[26] Mayhew L, Smith D An investigation into inequalities in adult lifespan. London: Cass Business School, 2016.

[27] Green M, Dorling D, Minton J The geography of a rapid rise in elderly mortality in England and Wales, 2014-15. Health Place 2017;44:77–85. 10.1016/j.healthplace.2017.02.002 28199896

