# *Correction: North-South disparities in English mortality 1965–2015: longitudinal population study*

**DOI:** 10.1136/jech-2017-209195corr1

**Published:** 2017-10-27

Buchan IE, Kontopantelis E, Sperrin M, *et al.* North-South disparities in English mortality 1965–2015: longitudinal population study. *J Epidemiol Community Health* 2017;71:928–936.

There is a mistake in the calculation of the estimate of aggregate deaths. On page 929, at the end of the ‘ premature mortality time trends’ section, the estimate:

‘1 173 360 (95% CI: 1 112 724 to 1 234 280)’

should read:

‘986 434 (95% CI: 943 234 to 1 029 121)’.

