# Supplementary material for: North-South disparities in English mortality1965–2015: longitudinal population study
Source: J Epidemiol Community Health. 2017 Aug 7;71(9):928–36. doi: 10.1136/jech-2017-209195 (PMC5561382; doi:10.1136/jech-2017-209195)
Supplement: Supplementary file 1 [file jech-2017-209195supp001.pdf]

Figure A1: Population age structure differences for the North and South of England for the periods 1965–1973 and 2010–2015.

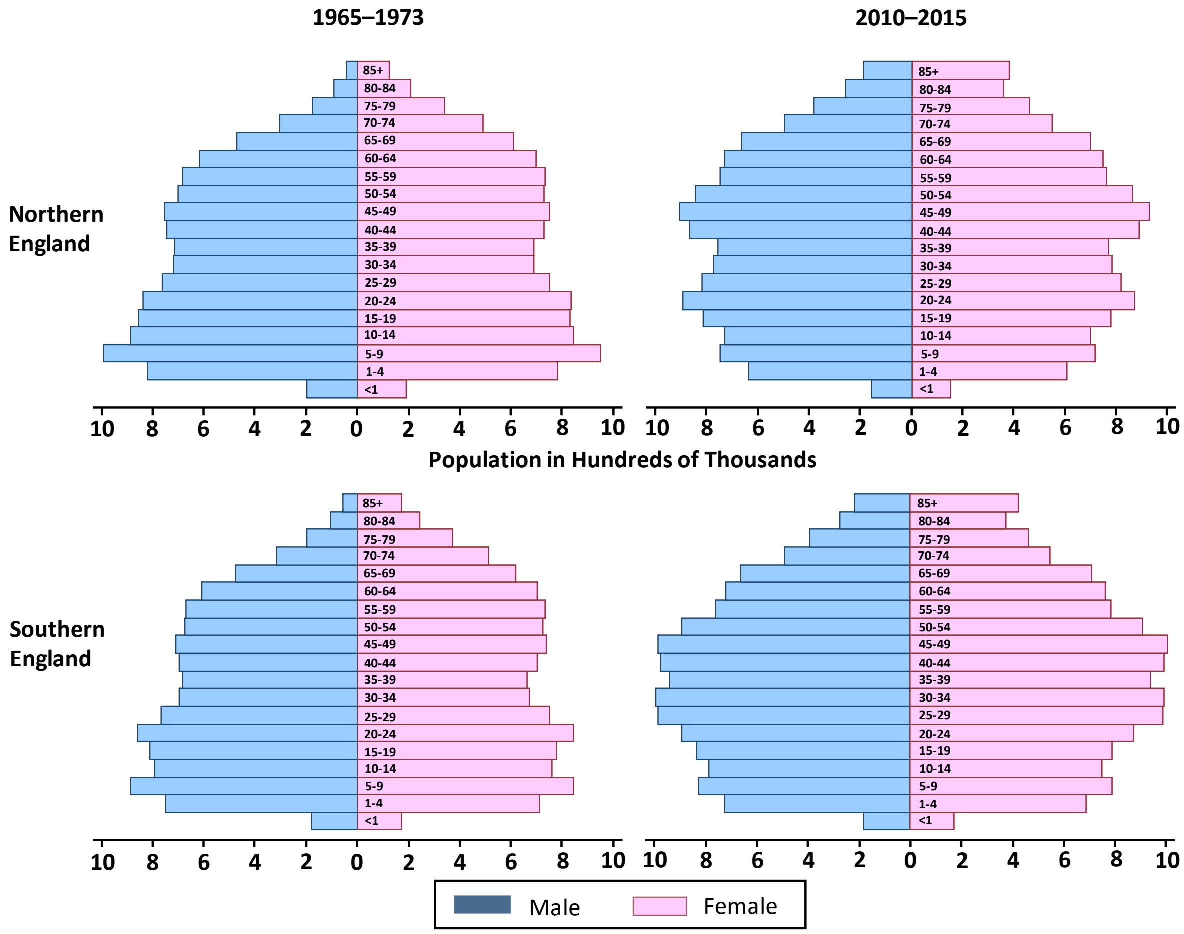

Figure A2: Excess premature (age <75 years) mortality in the North compared with South of England, adjusted for within-year age-sex differences.

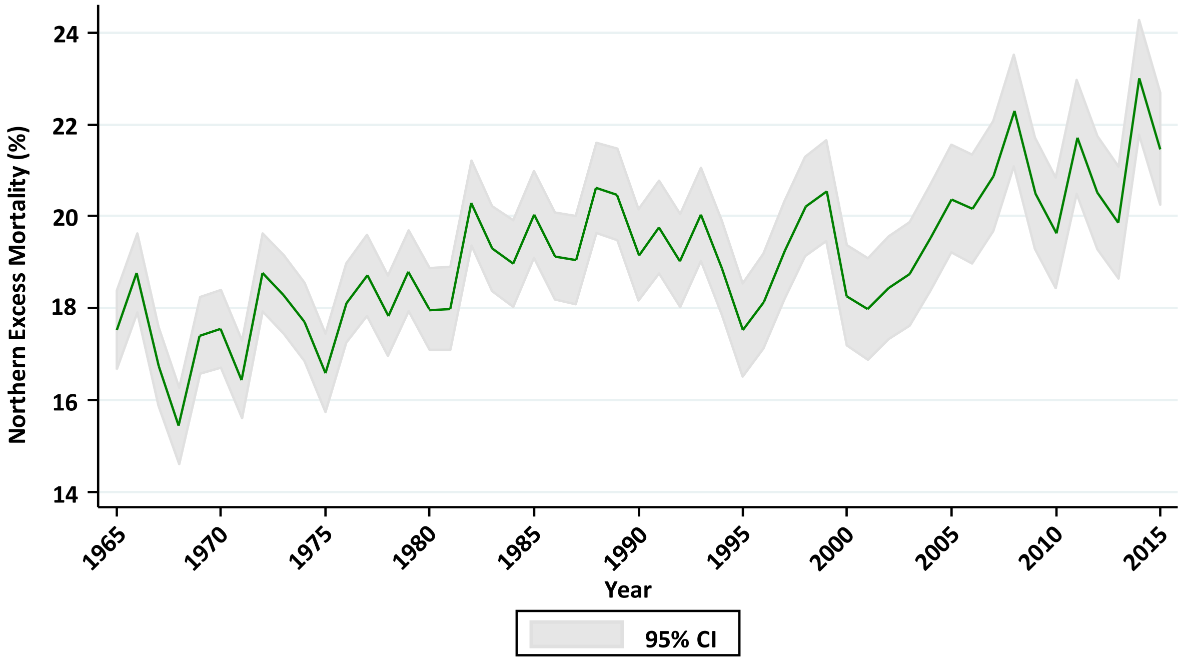

Table A1: Relative change in northern excess mortality in the year after the recession compared with three years earlier, for the recessions of 1973–75, 1980–81, 1990–91 and 2008–09.

Comparison	Estimate (95% CI)
1976 vs. 1973	0.998 (0.987–1.008)
1982 vs. 1979	1.012 (1.001–1.022)
1992 vs. 1989	0.988 (0.977–1.000)
2010 vs. 2007	0.989 (0.975–1.003)
